# Assessment of Hematological and Biochemical parameters with extended D-Ribose ingestion

**DOI:** 10.1186/1550-2783-5-13

**Published:** 2008-09-15

**Authors:** John Seifert, Angela Frelich, Linda Shecterle, John St Cyr

**Affiliations:** 1 Human Performance Laboratory, St. Cloud State University, St. Cloud, MN, USA; 2 Jacqmar, Inc., Minneapolis, MN, USA

## Abstract

D-ribose, a naturally occurring pentose carbohydrate, has been shown to replenish high- energy phosphates following myocardial ischemia and high intensity, repetitive exercise. Human studies have mainly involved short-term assessment, including potential toxicity. Reports describing adverse effects of D-ribose with prolonged ingestion have been lacking. Therefore, this study assessed the toxicity of extended consumption of D-ribose in healthy adults. Nineteen subjects ingested 20 grams/Day (10 grams, twice a Day) of ribose with serial measurements of biochemical and hematological parameters at Days 0, 7, and 14. No significant toxic changes over the 14-day assessment period occurred in complete blood count, albumin, alkaline phosphatase, gamma glutamyltransferase, alanine amiotransferase, and aspartate aminotransferase. However, D-ribose did produce an asymptomatic, mild hypoglycemia of short duration. Uric acid levels increased at Day 7, but decreased to baseline values by Day 14. D-ribose consumption for 14 days appears not to produce significant toxic changes in both hematological and biochemical parameters in healthy human volunteers.

## Background

D-ribose, a naturally occurring pentose carbohydrate, has shown benefits with negligible adverse effects. Pre-clinical studies have demonstrated the recovery benefits in depressed high-energy phosphates with D-ribose as well as functional improvements in the myocardium following ischemia and in isolated skeletal muscle [1-4]. Clinically, congestive heart failure patients have experienced improvements in their diastolic dysfunction, ventilatory efficiency, a better quality of life, and improved physical function activities when taking oral D-ribose [5-7]. The benefits in sports medicine have not been as obvious. When athletes were subjected to repetitive, high intensity exercise, Hellsten, et al. [8] reported that D-ribose provided a recovery benefit in depressed muscular high-energy phosphates in athletes subjected to repetitive, high intensity exercise. Others have also reported the benefits of D-ribose [9-13]. However, some studies did not find a beneficial role of D-ribose in sports medicine. The inconsistent results found in these studies could primarily be due to differences in study design [14-19]. Even so, ribose continues to attract the interest of athletes.

Safety of supplements is important and changes in serum biochemical and/or hematological markers could provide an early indication of cellular toxicity. Published studies on D-ribose have mainly centered on acute, short-term investigations, ranging from hours to a few days [2,4,8,9,11,20]. This study was designed to evaluate biochemical and hematological parameters in healthy adults who consumed D-ribose for 14 days.

## Methods

We investigated the potential toxicity in hematological and biochemical markers during a 14-day supplementation of oral D-ribose. St. Cloud State University's Institutional Review Board approved this study and informed, written consent was obtained from each subject prior to participation. Twenty-one healthy, non-diabetic, adult subjects (19–25 years of age) were initially enrolled; however, only 19 subjects (12 males, 7 females) completed the study. Each subject maintained their daily diet and routine exercise habits from interviews. Twenty grams of D-ribose, mixed in water, was consumed in two equally divided doses at breakfast and dinner mealtimes each day.

Venous blood samples were drawn from an antecubital vein and assessed for hematological and biochemical laboratory parameters at baseline and at Days 7 and 14 while on D-ribose. Each blood sample was collected at an early time period each day in a fasted state. The blood samples were collected in the AM, using a Becton Dickinson Vacutainer (Becton Dickinson, Inc., Franklin Lakes, NJ) coated with lithium heparin. All samples were collected at room temperature (23°C), and each tube was initially frozen before undergoing subsequent duplicate analyses. Measured parameters included: complete blood count (CBC, including hemoglobin (Hgb), hematocrit (Hct), white blood count (WBC), platelet counts (Plts), albumin, alkaline phosphatase (ALKP), gamma glutamyltransferase (GGT), alanine aminotransferase (ALT), aspartate aminotransferase (AST), uric acid, and glucose levels. Complete blood counts were analyzed using a Coulter Counter. Plasma samples were analyzed for albumin (Johnson & Johnson Vitros II), glucose (YSI 2300 Stat Analyzer), ALKP (Johnson & Johnson Vitros II), GGT (Johnson & Johnson Vitros II), ALT (Johnson & Johnson Vitros II), AST (Johnson & Johnson Vitros II), and uric acid (Uric Acid Kit, Sigma Chemicals) levels.

Measured parameters, including duplicate assessments, were analyzed by Analysis of Variance (ANOVA). Independent variables, such as gender, were also statistically analyzed by ANOVA. An accepted alpha level of significance was 0.05. Analyses (mean ± SD) were performed with MINITAB 12.1 (Minitab Incorporated, PA).

## Results

There were no adverse physical symptoms in the 19 subjects completing the study. There were no statistically significant differences from baseline to the Day 7 and 14 evaluations in all subjects for CBC parameters: Hgb, Day 7(p < 0.44), Day 14 (p < 0.31); Hct, Day 7 (p < 0.3), Day 14 (p < 0.41); WBC, Day 7 (p < 0.30), Day 14 (p < 0.44); Plts, Day 7 (p < 0.25), Day 14 (p < 0.40) (Table 1). Further, there were no significant differences between genders in measured CBC parameters. However, there was a slight decrease in WBC levels in all subjects with a greater decrease in females at Day 14.

**Table 1:** Hematological Parameters. (Mean ± SD with p value significance for all subjects)

	Hgb (grams/dL)	Hct (%)	WBC (10^3^/uL)	Plts (10^3^/uL)
Males				
Baseline	15.4 ± 0.56	45.4 ± 1.70	6.13 ± 0.82	235 ± 55.10
Day 7	15.1 ± 0.87	44.9 ± 0.90	6.29 ± 1.13	242 ± 62.41
Day 14	15.1 ± 0.69	44.7 ± 2.03	6.25 ± 1.06	246 ± 60.43
Females				
Baseline	13.3 ± 0.45	39.5 ± 1.07	6.22 ± 1.64	236 ± 36.81
Day 7	13.3 ± 0.52	39.7 ± 1.21	6.10 ± 0.82	230 ± 48.05
Day 14	13.5 ± 0.60	40.1 ± 2.21	5.69 ± 1.17	232 ± 31.96
All Subjects				
Baseline	14.3 ± 1.14	42.5 ± 3.26	6.18 ± 1.14	235 ± 48.03
Day 7	14.2 ± 1.17	42.2 ± 3.48	6.19 ± 1.01	236 ± 56.42
Day 14	14.3 ± 1.01	42.4 ± 3.09	5.97 ± 1.10	239 ± 51.18
p values				
At Day 7	p < 0.44	p < 0.39	p < 0.30	p < 0.25
At Day 14	p < 0.31	p < 0.41	p < 0.44	p < 0.40

**Figure 1:**
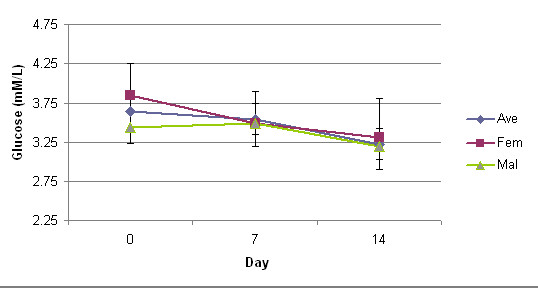
**Glucose levels over study duration**. bars represent standard deviation.

**Figure 2:**
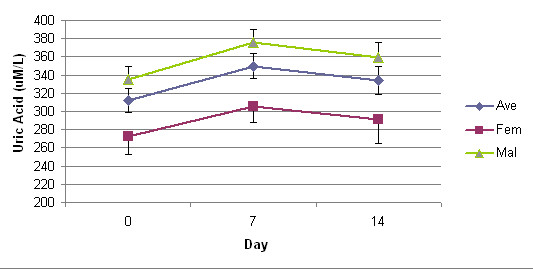
**Uric Acid levels over study duration**. bars represent standard deivation.

Albumin, ALKP, AST, ALT, GGT levels, with significance are presented in Table 2. Plasma glucose and uric acid levels are presented in figures 1 and 2. There were no significant differences from baseline compared to Days 7 and 14 values of plasma glucose levels (p < 0.41 and p < 0.14, respectively) and uric acid levels (p < 0.40 and p < 0.28, respectively). No significant differences were found at Day 7 in the ALKP (p < 0.41), GGT (p < 0.03), ALT (p < 0.06), AST (p < 0.11), and plasma glucose levels (p < 0.41). However, at Day 7 there were slight elevations from baseline values in AST and uric acid measurements. This observed elevation in both AST and uric acid levels at Day 7 rebounded towards baseline values by Day 14. Measured plasma glucose levels, even though not statistically significant at Day 7 (p < 0.41) and at Day 14 (p < 0.14), and fell over the 14-Day study period (3.64 ± 0.80, 3.55 ± 0.85, 3.23 ± 1.03 mM/L) without clinical symptoms.

**Table 2:** Liver Function Parameters. (Mean ± SD with p value significance for all subjects)

	ALB (grams/dL)	ALK P (U/L)	AST (U/L)	ALT (U/L)	GGT (U/L)
Males					
Baseline	5.077 ± 0.02	70.2 ± 20.06	21.3 ± 6.08	28.4 ± 6.79	27.6 ± 15.18
Day 7	5.087 ± 0.04	70.4 ± 19.74	19.6 ± 8.50	28.8 ± 6.55	22.7 ± 9.55
Day 14	5.078 ± 0.03	70.8 ± 20.41	25.6 ± 8.90	28.3 ± 11.79	25.4 ± 13.82
Females					
Baseline	5.065 ± 0.01	63.6 ± 12.23	12.4 ± 3.21	20.6 ± 5.74	14.9 ± 3.29
Day 7	5.075 ± 0.02	64.4 ± 9.00	19.6 ± 9.98	19.4 ± 7.98	15.0 ± 3.51
Day 14	5.076 ± 0.03	67.7 ± 10.44	15.7 ± 5.68	20.3 ± 6.24	15.1 ± 3.48
All					
Baseline	5.073 ± 0.02	66.9 ± 17.51	16.9 ± 6.75	24.5 ± 7.37	21.2 ± 13.57
Day 7	5.082 ± 0.03	67.4 ± 16.55	21.8 ± 9.06	24.1 ± 8.29	18.3 ± 8.62
Day 14	5.077 ± 0.02	69.2 ± 17.12	20.6 ± 9.11	24.3 ± 10.65	20.3 ± 12.11
p values					
At Day 7	p < 0.01	p < 0.41	p < 0.11	p < 0.06	p < 0.03
At Day 14	p < 0.27	p < 0.46	p < 0.11	p < 0.31	p < 0.08

Assessment of biochemical parameters between genders revealed no significant differences during the 14 days for both albumin (p < 0.27) and ALT (p < 0.31). There was no difference in ALKP in males; however, females demonstrated a slight increase at Day 14, which appeared to account for the noted increase in all subjects at the end of the study endpoint (Table 2). Measured GGT levels slightly decreased in males, unlike the slight increase found in females at Day 7; however these levels were comparable to baseline by Day 14. Measured AST levels demonstrated a potentially increasing trend (p < 0.11) throughout the study in males. Female AST values increased at Day 7 and fell by Day 14.

Uric acid levels were elevated without significance (p < 0.40) in all subjects at Day 7 with females demonstrating less of an increase than males. However, the observed elevated uric acid levels in all subjects at Day 7 declined by Day 14. Both genders demonstrated a decrease in serum glucose levels over the study.

## Discussion

The safety of oral D-ribose has been reported in acute settings in humans with no significantly adverse effects; however, a drop in plasma glucose levels is common. Segal and Foley [21,22] found different levels of hypoglycemia with D-ribose supplementation depending upon dose with higher doses producing a greater decrease. Gross et al. [23] has published similar findings. In nine healthy subjects (8 males, 1 female, 23–37 years of age), D-ribose doses of 83.3, 166.7 and 222.2 mg/kg/hr were given for at least 4 hours, both orally and intravenously, with serum ribose levels increasing in a dose-dependent manner (maximum concentration of 75–85 mg/dl). A 166.7 mg/kg/hr of D-ribose resulted in a 25% reduction in measured glucose levels (51.9 – 56.8 mg/dl) with an increase in serum insulin concentration (mean: 8.4 to10.4 uU/ml). The variations in insulin levels were acute; and did not solely account for the persistent hypoglycemia. Fenstad et al. [24] concurred that D-ribose's affected on plasma glucose values was dose dependent. Oral D-ribose doses of 5 and 10 grams produced an acute state of hypoglycemia (mean values: 3.08 and 2.77 mmol/L, respectively), which returned to baseline values approximately 2 hours post consumption. Insulin levels revealed an initial increased spike (3.0–3.6 uIU), peaking at 15 min post ingestion, and returning to relative baseline values by 45–60 min post consumption. Our study involving the extended supplementation of D-ribose concurs with previous published reports that even a longer duration of oral consumption results in a state of asymptomatic hypoglycemia.

Fenstad et al. [24] also found that D-ribose (5 and 10 grams/oral dose) produced an increase in uric acid levels. In our study involving a longer consumption period, elevated levels of uric acid, peaking at Day 7 and then trending toward baseline by Day 14 were observed. Uric acid production occurs due to the synthesis of purine nucleotides, degraded through regulated reactions. The net increase in uric acid levels may result from an increase in de novo purine synthesis with a concomitant enhancement in purine nucleotide degradation [25]. Yamaoka and Itakura [26] reported that purine nucleotide biosynthesis is regulated by amidophosphoribosyltransferase and phosphoribosyl-pyrophosphate synthetase in the de novo pathway. Accelerated biosynthesis, catabolism of purine nucleotides or decrease in urinary excretion, could all potentially account for this hyperuricemia. Further, ribose combining with adenine, producing adenosine, could enhance catabolism with increasing production of uric acid when ingesting ribose.

Individuals have occasionally experienced mild gastrointestinal symptoms, such as nausea, loose stools, or rare bouts of diarrhea [20,27]. Pliml et al. [20] stated that some patients experienced mild gastrointestinal symptoms, such as nausea and diarrhea; although a dose of 60 grams/day was used in that study. Our subjects did not experience any gastrointestinal problems with a dose of 20 grams per day. Recommended doses for D-ribose in the literature range between 5–7 grams and the 10 gram dose in our study did not produce any adverse gastrointestinal maladies.

There are few reports on adverse laboratory values with D-ribose in animals. D-ribose (0, 5, 10, 20%) fed to Wistar rats for 13 weeks revealed a significant decrease in cholesterol, triglycerides and phospholipid levels with significant increases in liver function tests of alkaline phosphatase, aspartate aminotransferase, and albumin/globulin ratio, without abnormal histopathological findings [28]. Griffiths et al. [28] also reported no significant changes in measured CBC parameters; however, there was a significant increase in neutrophils with a significant reduction in lymphocytes. We found no significant abnormal hepatic values or significant abnormalities in hematological parameters with extended oral consumption of D-ribose in healthy individuals.

## Conclusion

In summary, 20 grams of oral D-ribose/day for 14-days in healthy subjects did not elicit significant adverse hematological or biochemical abnormalities. However, a mild state of hypoglycemia and hyperuricemia can be observed after oral consumption.

## Competing interests

JS, AF and LS have no competing interests. JSC is a part time consultant for Bioenergy, Inc., a ribose company. Bioenergy, Inc. did not provide any support for this study.

## Authors' contributions

JS carried out research, oversaw project and helped write the manuscript. AF carried out the research. LS data analysis and writing. JSC writing and data interpretation. All authors have read and approved the final manuscript.

## Acknowlegements

We would like to acknowledge Lisa Gleason for her help in manuscript preparation and all of the subjects who participated in this study.
